# Extended High Frequency Hearing, but Not Tinnitus, Is Associated With Every-Day Cognitive Performance

**DOI:** 10.3389/fpsyg.2022.913944

**Published:** 2022-06-14

**Authors:** Sebastian Waechter, Wayne J. Wilson, Måns Magnusson, K. Jonas Brännström

**Affiliations:** ^1^Department of Logopedics, Phoniatrics and Audiology, Lund University, Lund, Sweden; ^2^School of Health and Rehabilitation Sciences, University of Queensland, Brisbane, QLD, Australia; ^3^Department of Otorhinolaryngology, Lund University, Lund, Sweden

**Keywords:** tinnitus, extended high frequency, hearing loss, cognition, perceived exertion, visual task

## Abstract

Research into the potential associations between tinnitus and cognition has investigated specific cognitive domains in laboratory settings despite adults with tinnitus reporting broad cognitive difficulties in every-day life. To address this limitation, the present study compared performance and perceived exertion on a visual office-like task in 38 adults with tinnitus (19 with normal hearing and 19 with hearing loss) and 38 adults without tinnitus (19 with normal hearing and 19 with hearing loss) matched for age, sex and educational background. All participants were also assessed for hearing, anxiety and depression, and participants with tinnitus were also assessed for tinnitus handicap. No associations were found between presence of tinnitus and cognitive performance (mean total rate correct score on the visual office-like task being 2.9 for the tinnitus group, 2.8 for the control group, *p* = 0.612) and perceived exertion (mean ratings of perceived exertion on the Borg CR10-scale being 5.8 for the tinnitus group, 6.5 for the control group, *p* = 0.063) on the visual office-like task when corrected for standard (0.125 to 8 kHz) and extended high frequency (10 to 16 kHz) hearing thresholds, anxiety, and depression. The correction for extended high frequency average (10, 12.5, 14, and 16 kHz) hearing threshold was significant for performance (*p* = 0.009) but not perceived exertion on the visual office-like task. Overall, the results showed extended high frequency hearing, but not tinnitus, was associated with every-day cognitive performance. This indicates clinical testing of hearing thresholds above 8 kHz could support clinicians’ identification and management of cognitive difficulties. One management method suggested by the current findings would include provision of auditory stimulation at frequencies exceeding the frequency response of many current hearing aids.

## Introduction

A common complaint among adults with tinnitus is that tinnitus reduces their ability to concentrate in every-day life (Watts et al., 2018). Such complaints appear to be supported by laboratory findings that adults with tinnitus perform poorer than control participants without tinnitus on behavioral measures of executive attention, selective attention and working memory (Mohamad et al., 2016). This is seen even when accounting for associations between tinnitus and anxiety and depression, which may be confounding factors in the association between tinnitus and cognitive performance (Kaiser et al., 2003; Cisler and Koster, 2010; Peckham et al., 2010; McCormack et al., 2015; Mohamad et al., 2016).

Recently, research associating tinnitus and cognition has been challenged by its failure to control for hearing and hearing impairment (Mohamad et al., 2016). The combination of high prevalence of hearing impairment among tinnitus patients (Sanchez et al., 2005; Oosterloo et al., 2021), clear associations between hearing impairment and cognitive decline (Uchida et al., 2019), and tendencies among tinnitus patients to over-associate hearing difficulties to their tinnitus rather than their hearing impairment (Henry et al., 2015), indicates hearing status as being a possible confounding factor that must be considered when evaluating cognitive performance in tinnitus patients (Clarke et al., 2020). This indication has been supported by recent studies finding no association between tinnitus and cognitive performances after controlling for hearing loss at typically measured frequencies (Waechter and Brännström, 2015; Waechter et al., 2019, 2021; Glick and Sharma, 2020; Hamza and Zeng, 2021; Jensen et al., 2021; although exceptions are noted: Sherlock and Brungart, 2021) and at extended high frequencies (Waechter et al., 2019, 2021; Jensen et al., 2021).

A further issue clouding the association between tinnitus and cognition is the lack ecological validity resulting from patient reports of tinnitus affecting concentration in every-day life compared to researchers who assess tinnitus effects using isolated cognitive tasks in the laboratory. The need for future studies to investigate whether tinnitus is associated with cognitive difficulties in an everyday life context has been highlighted by several research groups (McKenna et al., 2014; Waechter and Brännström, 2015; Neff et al., 2021). In every-day life, cognitively challenging situations present in complex ways requiring the adult with tinnitus to successfully complete multiple, interacting cognitive functions over prolonged periods of time. Such complex cognitive processing is not well reflected in research investigating single cognitive functions (such as short-term elements or memory or attention) in isolation over short periods of time in controlled, laboratory conditions.

Using more ecologically valid measures of cognitive performance could provide better understanding of how tinnitus affects cognition in daily life, particular if the cognitive tasks used more closely resemble tasks performed in real-life. An example of such a task was reported by Hua et al. (2014) as an office-like task requiring adults to use business-related information presented in written tables to answer 32 questions about business performance. This task was originally used to study the possible impact of auditory noise on work related performance. Hua et al. (2014) found no strong correlations between performance on their office-like task and any one, specific cognitive domain. The authors concluded that performance on their office-like task required adequate functioning across multiple cognitive domains, as would be expected in the completion of cognitive tasks in the real-world.

The ecological validity of measures of cognition could also be improved by adding measures of exertion (also called effort) to the commonly used measures of performance. Previous studies exploring tinnitus and cognitive performance have focused solely on cognitive performance, typically by measuring accuracy and reaction time on specific cognitive tasks (Mohamad et al., 2016). Such performance measures disregard the potentially high levels of exertion in adults who need to suppress their tinnitus to be able to perform their cognitive tasks, especially if this suppression must be maintained for long periods in each working day.

The present study aimed to provide a more ecologically valid investigation of tinnitus and cognition in adults with and without hearing impairment by examining behavioral performances and perceived exertion on a visual office-like task (VOLT; Hua et al., 2014). Two research questions were considered: (1) is tinnitus associated with performance on the VOLT after correcting for hearing thresholds, anxiety and depression; and (2) is tinnitus associated with degree of perceived exertion on the VOLT after correcting for hearing thresholds, anxiety and depression?

## Materials and Methods

### Participants

Seventy-six adult volunteers were purposely sampled through audiological clinics and public advertising in southern Sweden. Thirty-eight adults, School of Health and Rehabilitation Sciences, were recruited into the tinnitus group (TG) on the basis of subjectively reporting constant or fluctuating tinnitus in the last 6 months or longer prior to their participating in the study. Thirty-eight adults, 19 with and 19 without hearing impairment, were recruited into the control group (CG) on the basis of not experiencing tinnitus and being matched to TG participants for education (all were either current or former university students), age (matched participants differed in age by 24 months or less) and sex. While hearing status was not included as a matching criterion, the TG and CG showed similar audiograms and did not differ significantly in terms of hearing thresholds at frequencies 0.5 to 4 kHz or 10 to 16 kHz. For all participants, normal hearing was defined as worse ear hearing thresholds of 20 dB HL or better at all tested frequencies from 0.125 to 8 kHz (see specifications below), and hearing impairment was defined as having at least one hearing threshold worse than 20 dB HL in the better ear at the mentioned frequencies. All participants reported normal or corrected to normal vision and were fluent in Swedish but not necessarily native Swedish speakers. Table 1 shows the descriptive statistics for all participants, and Table 2 shows the tinnitus characteristics for the participants in the TG. Eighty-two participants were initially recruited, however, six were excluded due to difficulty finding age matched co-participants.

**TABLE 1:** Demographic statistics for all participants and separately by group.

			All participants	Tinnitus group (TG)	Control group (CG)
Participants		N	76	38	38
Sex		Male/Female	32/44	16/22	16/22
Age (years)		Range	23.3 to 66.3	23.3 to 65.2	23.7 to 66.3
		Mean ± SD	36.8 ± 12.3	36.9 ± 12.4	36.8 ± 12.4
Hearing status	S-PTA (0.5 to 4 kHz)	Range	–5.0 to 73.1	–4.4 to 53.1	–5.0 to 73.1
		Mean ± SD	17.8 ± 19.1	16.0 ± 17.3	19.7 ± 20.8
	EHF-PTA (10 to 16 kHz)	Range	–7.5 to 75.0	–3.8 to 73.8	–7.5 to 75
		Mean ± SD	30.0 ± 24.6	31.7 ± 24.3	28.2 ± 25.1
Hospital Anxiety and Depression Scale (HADS) scores	Anxiety score (HADSA)	Range	0 to 17	1 to 17	0 to13
		Mean ± SD	6.8 ± 3.4	7.3 ± 3.7	6.4 ± 3.1
	Depression score (HADSD)	Range	0 to 11	0 to 9	0 to11
		Mean ± SD	4.1 ± 2.9	3.7 ± 2.7	4.5 ± 3.1

**TABLE 2:** Characteristics of participants with tinnitus (*n* = 38).

Self-reported cognitive difficulties due to tinnitus		yes = 31, no = 7
Tinnitus Handicap Inventory (THI) score	Range	6 to 84
	Mean ± *SD*	35.3 ± *21.4*
Tinnitus severity according to Tinnitus Handicap	No handicap	11
Inventory (THI) score	Mild handicap	8
	Moderate handicap	13
	Severe handicap	6
Time since tinnitus onset (years)	Span	0.5 to 59.9
	Mean ± SD	9.9 ± *11.3*

The present study was approved by the Regional Ethical Review Board in Lund, Sweden (approval number 2014/95). All participants were informed about the purpose and conditions of the study prior to participating and gave written consent to participate.

### Equipment

Pure tone audiometry was performed using a Madsen Astera2 (GN Otometrics) audiometer with HDA 200 (Sennheiser) earphones. A Brüel & Kjær type 2209 sound level meter and type 4153 artificial ear were used to calibrate the audiometer in accordance with ISO 389-8 (2004) and ISO 389-5 (2006).

### Audiometry

Pure tone hearing thresholds were measured in each ear at frequencies of 0.125, 0.25, 0.5, 1, 1.5, 2, 3, 4, 6, 8, 10, 12.5, 14 and 16 kHz according to ISO 8253-1 (2010), using a two-down/one-up (–10dB/+5dB) adaptive method. From these thresholds, mean pure tone average thresholds at standard frequencies (0.5, 1, 2 and 4 kHz; S-PTA) and extended high frequencies (EHF: 10, 12.5, 14 and 16 kHz; EHF-PTA) were calculated for each participant. Thirteen of the 76 included participants (7 tinnitus participants, 6 control participants) did not respond to the highest sound intensity tested at one or more frequencies at 10 to 16 kHz. The highest tested dB HL levels at unheard frequencies were used to calculate EHF-PTA for those participants.

### Behavioral Cognitive Task – Visual Office-Like Task

The visual office-like task (VOLT) presented by Hua et al. (2014) as an unnamed “work-related task” was used as the “real-world” measure of cognitive performance. The VOLT consists of 32 subtasks each consisting of an information table (15 rows, 6–7 columns) and a question to answer. Sixteen subtasks have questions requiring evaluation of information from only two columns to give a correct answer (e.g., “Which car costs the most?”), while remaining 16 subtasks have questions requiring evaluation of information from four different columns to give a correct answer (e.g., “Which state ruled by CDU, and accounting for 1.96 % or less of Germanys BNP, has the most workers within the construction sector?”). The next subtask is presented as soon as the participant has used the computer keyboard to give an answer (specifically: typing the number of the row corresponding with the row thought to be the correct answer to the subtask question) or when the maximum subtask duration (60 s) has been reached. Hereafter, the VOLT subtask requiring evaluation from two columns will be referred to as the simple task and the VOLT subtask requiring evaluation from four columns will be referred to as the complex task. All VOLT subtasks together will be referred to as the total task.

### Questionnaires and Scales

#### Borg CR10-Scale

The Borg CR10-scale (Borg, 1990) was used to assess the participants’ perceived exertion when performing the VOLT. This scale provides a rating of perceived exertion (PE) with high test retest reliability (r > 0.8; Borg, 2007). The Borg CR10-scale is a hybrid category (C) and ratio (R) scale of exertion where numbers (making it a ratio scale) are presented vertically along with describing words (making it a categorical scale), ranging from zero (0, “none at all”) to ten (10, “extremely strong”). The responder can also indicate exertion greater than ten by ticking a “maximal exertion” option, which was scored as eleven (11) in the present study. This choice was made by the authors of the present article in the absence of guidance on how to score responses indicating “maximal exertion” in the original publication by Borg (1990). The scale was originally developed to assess exertion on physical tasks, but has been adopted in hearing research in order to assess aspects such as perceived effort in speech processing tasks (Larsby et al., 2005; Brännström et al., 2018), perceived sound level at work (Kähäri et al., 2003), and perceived effort when performing VOLT in noise and in quiet (Hua et al., 2014).

#### Hospital Anxiety and Depression Scale

The Hospital Anxiety and Depression Scale (HADS) (Zigmond and Snaith, 1983) is a screening questionnaire for symptoms of anxiety and depression in adult patients. The questionnaire has shown good validity and test-retest variability (Hermann, 1997; Bjelland et al., 2002) and is frequently used in both clinical and research applications. The HADS consists of 14 statements, seven forming an anxiety subscale and seven forming a depression subscale. The patient is asked to respond to each statement by ticking one out of four response options based on what response is closest to how they have been feeling the past week. Response options are scored 0-3 from lowest to highest degree of anxiety/depression [e.g., “I feel cheerful” with response options of “Definitely” (0 points) to “Not at all” (3 points)]. Responses are scored to provide a HADS anxiety (HADSA) score ranging from 0 to 21, a HADS depression (HADSD) score ranging from 0 to 21, and a total HADS score ranging from 0 to 42. Subscale scores of 11 or more indicate clinically significant symptoms of anxiety/depression. In the present study, the validated Swedish version of the HADS was used (Lisspers et al., 1997).

#### Tinnitus Handicap Inventory

The Tinnitus Handicap Inventory (THI: Newman et al., 1996) is a questionnaire for assessing degree of perceived handicap due to tinnitus (also referred to as tinnitus severity). It has shown good validity and test-retest variability (McCombe et al., 2001) and is one of the most common methods of assessing tinnitus severity in both clinical and research applications. It consists of 25 questions (e.g., “Does your tinnitus make you angry?”) with response options of “Yes” (4 points), “Sometimes” (2 points), or “No” (0 points). Scores are summed to obtain a total THI score ranging from 0 to 100, where scores of 0 to 16 indicate no tinnitus handicap, 18 to 36 indicate mild tinnitus handicap, 38 to 56 indicate moderate tinnitus handicap, and 58 to 100 indicate severe tinnitus handicap (Newman et al., 1998). In the present study, the validated Swedish version of the THI was used (Müller et al., 2016).

### Procedure

All but two participants completed the testing in the following order in a sound treated room complying with the maximum permissible ambient sound levels stated in ISO 8253-1 (2010):

(1) Otoscopy, and pure tone audiometry at 0.125-16 kHz in each ear;
(2) cognitive testing (reported in Waechter et al. (2021)),
(3) ratings of PE pre-VOLT (baseline rating of PE prior to the VOLT). Specifically, participants were asked to “Please indicate the level of exertion you are experiencing right now”;
(4) the VOLT;
(5) ratings of PE due to the VOLT. Specifically, participants were asked to “Please indicate the level of exertion experienced due to the visual office-like task”;
(6) the HADS; and
(7) the THI and a short interview about the participant’s tinnitus (participants in the TG only).

Note that it was emphasized to the participants that they should rate their degree of exertion, not their degree of performance or their perception of task difficulty. Two participants underwent the test battery in a slightly different order (completing audiometry last instead of first in the procedure) due to administrative difficulties.

### Data Analysis

As both accuracy and response time were needed for successful completion of VOLT, and analyzing those factors separately could lead to incorrect conclusions regarding task performance, these two measures were combined into a single factor. This factor was obtained by calculating the Rate Correct Score (RCS: Woltz and Was, 2006);


$$R\,C\,S=\frac{c}{\sum R\,T}$$

where c = number of correct responses, and RT = response time in seconds of all responses. Hereafter, the RCS will be referred to as task performance.

Data were analyzed descriptively by calculating skewness and kurtosis and inspecting frequency histograms and normal Q–Q plots. The equality of covariance matrices in the MANCOVA models were tested using the Box test, and the standardized residuals in these models were examined for normality of distribution and association with predicted standardized residuals. No observed findings threatened the assumptions for MANCOVA analyses.

The task performance results were analyzed using a one-way MANCOVA with the dependent variables being simple task-, complex task-, and total task-performance, the independent variable being presence of tinnitus, and the co-variables being HADSA score, HADSD score, S-PTA and EHF-PTA. The task performance results were re-analyzed first using worst ear hearing thresholds, and second using best hearing thresholds, instead of mean thresholds for both ears. As these analyses yielded similar results and thus did not change the interpretation of the findings, only the mean ear hearing threshold data and analysis are reported here. Similar results were expected when using mean, worst, and best hearing thresholds, as the vast majority of included participants had symmetrical hearing.

The ratings of PE results were analyzed using a one-way ANCOVA with the dependent variable being absolute PE due to VOLT, the independent variable being presence of tinnitus, and the co-variables being HADSA score, HADSD score, S-PTA and EHF-PTA. The PE results were also analyzed using a using a one-way ANCOVA with the dependent variable being added PE due to VOLT (i.e., absolute PE due to VOLT minus baseline PE), the independent variable being presence of tinnitus, and the co-variables being HADSA score, HADSD score, S-PTA and EHF-PTA. The reason for analyzing added PE due to VOLT, in addition to absolute PE due to VOLT, was to control for baseline exertion. As the two analyses of PE yielded similar between-group (TG versus CG) results and thus did not change the interpretation of the findings, only the first PE data and analysis are reported here.

Pearson’s correlation between absolute PE and task performance were performed to analyze possible correlations between subjective degree of exertion and behavioral task performance.

In order to explore potential associations between tinnitus severity and VOLT performance or PE, four further Pearson’s correlations were performed, three between THI score and simple, complex and total VOLT performance, respectively, and one between THI score and PE.

The MANCOVA results motivated further exploration between EHF-PTA and VOLT performances. Partial correlations between EHF-PTA and simple, complex and total VOLT performances were performed controlling for age. The choice to control for age was done as it may be a confounding factor in the relationship between high frequency hearing and cognitive performance, indicated by researchers having reported age to be significantly associated with both poorer cognitive performance (Bialystok and Craik, 2006; Harada et al., 2013) and poorer hearing thresholds above 8 kHz (Jilek et al., 2014).

All statistical analyses were performed using IBM SPSS Statistics version 24.0.0.0, 64-bit edition for Windows (IBM Corp., 2016). An alpha level of 0.05 was used for significance for all analyses.

## Results

Table 3 shows means, ranges and standard deviations for task performance and perceived exertion, for all participants and groups separately.

**TABLE 3:** Scores [Rate Correct Scores (RCS) multiplied by 100] and ratings of perceived exertion on the VOLT, for all participants and separately by experimental group.

VOLT		All participants (*n* = 76)	Tinnitus group (TG; *n* = 38)	Control group (CG; *n* = 38)
Simple task performance	Range	2.1 to 10.6	2.1 to 9.5	3.4 to 10.6
	Mean ± *SD*	5.9 ± *1.8*	6.1 ± *1.9*	5.8 ± *1.7*
Complex task performance	Range	0.2 to 3.6	0.2 to 3.3	0.6 to 3.6
	Mean ± *SD*	1.6 ± *0.7*	1.6 ± *0.7*	1.6 ± *0.8*
Total task performance	Range	0.9 to 4.8	0.9 to 4.7	1.4 to 4.8
	Mean ± SD	2.8 ± *0.9*	2.9 ± *0.9*	2.8 ± *0.9*
Ratings of perceived exertion (PE) due to VOLT	Range	2 to 11	2 to 10	3 to 11
	Mean ± SD	6.2 ± *2.1*	5.8 ± *2.1*	6.5 ± *2.1*

**FIGURE 1:**
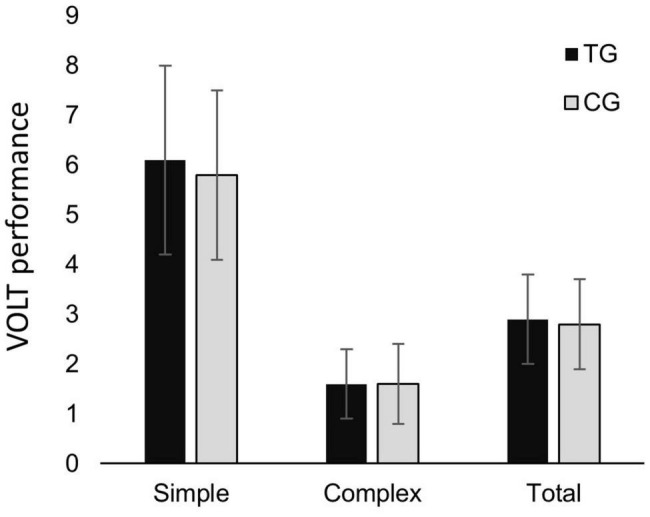
Visual office-like task (VOLT) performance (rates correct score; RCS) in simple VOLT subtask, complex VOLT subtask and total VOLT, in tinnitus group (TG) and control group (CG) respectively. Error bars depict standard deviations.

Table 4 shows the results of the multivariate MANCOVA analysis for task performance. The presence of tinnitus was not related to task performance [simple score (tinnitus mean: 6.1, control mean: 5.8), complex score (tinnitus mean: 1.6, control mean: 1.6) and total score (tinnitus mean: 2.9, control mean: 2.8); *p* = 0.612] when corrected for S-PTA, EHF-PTA, anxiety, and depression. The correction for EHF-PTA was significant (*p* = 0.009, partial η^2^ = 0.154). Table 5 shows the results of the univariate MANCOVA analyses for task performance. The presence of tinnitus was not related to task performance in the separate task conditions (simple, complex, or total task score). The correction for EHF-PTA was significant in each of test conditions [*p* < 0.05 for simple (partial η^2^ = 0.063), complex (partial η^2^ = 0.148), and total task (partial η^2^ = 0.139) score], indicating an association between better high-frequency hearing thresholds and higher task scores. The null hypothesis of equal covariance matrices was not rejected for the MANCOVA, with the Box’s M p-value being.416. Figure 1 shows the VOLT performances in the TG and the CG.

**TABLE 4:** Results of the multivariate MANCOVA analysis for the effect of tinnitus on VOLT performance (simple, complex and total task score combined), corrected for anxiety, depression, and hearing status [S-PTA (0.5 to 4 kHz); EHF-PTA (10 to 16 kHz)].

	F (3,68)	Pillai’s Trace	partial η^2^	*p*
Tinnitus	0.608	0.026	0.026	0.612
Hospital Anxiety and Depression Scale Anxiety score (HADSA)	0.106	0.005	0.005	0.956
Hospital Anxiety and Depression Scale Depression score (HADSD)	0.168	0.007	0.007	0.918
Mean hearing threshold at standard audiometric frequencies (S-PTA)	0.987	0.042	0.042	0.404
Mean hearing threshold at extended hearing frequencies (EHF-PTA)	4.128	0.154	0.154	0.009

**TABLE 5:** Results of the univariate MANCOVA analyses for the effect of tinnitus on VOLT score separately (simple, complex, and total task score), corrected for anxiety, depression, and hearing status [S-PTA (0.5 to 4 kHz); EHF-PTA (10 to 16 kHz)].

	VOLT	F (1, 70)	partial η^2^	*p*
Tinnitus	Simple	0.897	0.013	0.347
	Complex	0.262	0.004	0.611
	Total	0.895	0.013	0.347
Hospital Anxiety and Depression Scale Anxiety score (HADSA)	Simple	0.065	0.001	0.800
	Complex	0.020	< 0.001	0.887
	Total	0.009	< 0.001	0.925
Hospital Anxiety and Depression Scale Depression score (HADSD)	Simple	0.443	0.006	0.508
	Complex	0.306	0.004	0.582
	Total	0.390	0.006	0.534
Mean hearing threshold at standard audiometric frequencies (S-PTA)	Simple	0.042	0.001	0.838
	Complex	1.618	0.023	0.208
	Total	0.535	0.008	0.467
Mean hearing threshold at extended hearing frequencies (EHF-PTA)	Simple	4.726	0.063	0.001
	Complex	12.200	0.139	0.033
	Total	11.271	0.148	0.001
				

**FIGURE 2:**
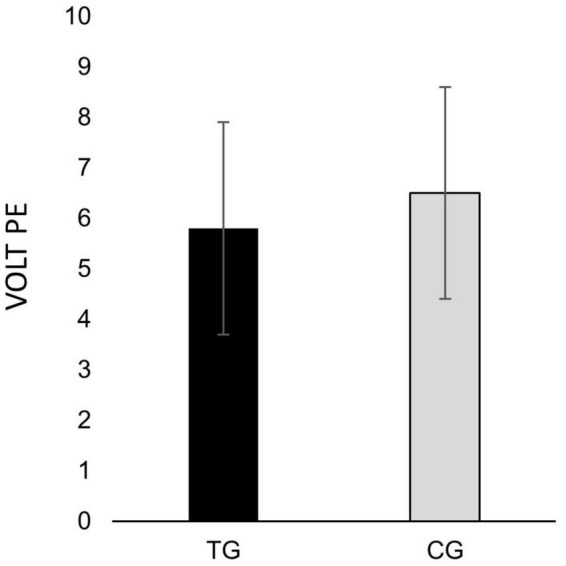
Subjective rating of perceived exertion (PE) due to performing the visual office-like task (VOLT), as rated by the Borg CR10-scale, in tinnitus group (TG) and control group (CG) respectively. Error bars depict standard deviations.

Table 6 shows the results from the ANCOVA analysis for PE due to the VOLT. The presence of tinnitus was not related to PE due to the VOLT (tinnitus mean: 5.8, control mean: 6.5; *p* = 0.063) when corrected for S-PTA, EHF-PTA, anxiety, and depression. In this model, none of the corrections reached significance. Figure 2 shows the PE ratings for the TG and the CG.

**TABLE 6:** Results of the ANCOVA analysis for the effect of tinnitus on ratings of perceived exertion, corrected for anxiety, depression, and hearing status [S-PTA (0.5 to 4 kHz); EHF-PTA (10 to 16 kHz)].

	F (3, 68)	partial η^2^	*p*
Tinnitus	3.578	0.049	0.063
Hospital Anxiety and Depression Scale Anxiety score (HADSA)	2.683	0.037	0.106
Hospital Anxiety and Depression Scale Depression score (HADSD)	0.006	< 0.001	0.940
Mean hearing threshold at standard audiometric frequencies (S-PTA)	0.892	0.012	0.366
Mean hearing threshold at extended hearing frequencies (EHF-PTA)	1.806	0.025	0.183
			

Pearson’s correlation indicated no significant association between absolute PE due to the VOLT and total task performance [*r*(75) = –0.129, *p* = 0.267]. This was also found when analyzing data from the TG [*r*(37) = 0.012, *p* = 0.944] and CG [*r*(37) = –0.259, *p* = 0.116] separately.

When analyzing data from individuals with tinnitus, Pearson’s correlation indicated no significant association between THI score and VOLT performance for any task condition [simple: *r*(37) = –0.237, *p* = 0.152, complex: *r*(37) = –0.038, *p* = 0.821, total: *r*(37) = –0.134, *p* = 0.424], or between THI score and PE [*r*(37) = 0.137, *p* = 0.412].

Significant negative partial correlations were found between EHF-PTA and VOLT performance for all task conditions [simple: *r*(75) = –0.337, *p* = 0.042, complex: *r*(75) = –0.325, *p* = 0.05, total: *r*(75) = –0.365, *p* = 0.026] when controlling for age.

## Discussion

The results of the present study indicated: (1) no significant association between tinnitus and VOLT performance (regardless of task complexity) when controlling for anxiety, depression and hearing thresholds, with the correction for EHF-PTA being significant for all task conditions; and (2) no significant association between tinnitus and PE on the VOLT when controlling for anxiety, depression and hearing thresholds. In addition, significant but weak correlations were found between EHF-PTA and VOLT performance (regardless of task complexity), when controlling for age. When analyzing data from individuals with tinnitus only, tinnitus severity was not significantly correlated with VOLT performance (regardless of task complexity) or PE.

Given the common complaints among adults with tinnitus regarding cognitive difficulties in every day-life, it was somewhat surprising to find no association between tinnitus and VOLT performance or PE in the present study. One interpretation of this finding is tinnitus does not relate to performance or perceived exertion in office-like tasks. Such an interpretation would be partly consistent with Mohamad et al. (2016) who concluded the evidence for tinnitus affecting individual cognitive functions was mixed for working memory, alerting attention, selective attention and executive attention, and not compelling for sustained attention. Given the mixed effects of tinnitus on individual cognitive tasks, it would be reasonable to expect similarly mixed effects of tinnitus on broader cognitive tasks such as the VOLT used in the present study. An interesting detail is this finding was the absence of an association between tinnitus and performance and PE despite a clear majority (31 of 38) of participants with tinnitus subjectively reporting difficulties with concentration according to their THI responses. This suggests a discrepancy between perceived versus actual effects of tinnitus on cognitive ability in the TG. It should be noted that the present findings of lack of difference between TG and CG on an office-like task demanding involvement of several cognitive domains to be successfully solved are in line with the findings of Cardon et al. (2019). They reported that general cognitive performances (as measured by a cognitive battery consisting of subtests of immediate and delayed memory, visuospatial capabilities, language, and attention) did not differ between tinnitus and control subjects when controlling for hearing thresholds (0.125–8 kHz), gender, age, and educational level. Cardon et al. (2019) did, however, find a tinnitus related effect on verbal fluency, which warrants further investigation.

An alternative explanation for the present study’s findings could be the VOLT not being sufficiently complex to trigger any cognitive difficulties or induce any fatigue in the participants with tinnitus. Such a possibility was supported by Waechter et al. (2021) who found the association between tinnitus and working memory neared significance as the complexity of the working memory task increased. This alternative explanation is challenged by the participants of the present study reporting strong to very strong perceived exertion during the VOLT (an average rating of 6 on the Borg CR10-scale). The use of more difficult office-like tasks capable of inducing more fatigue could require the task information to be presented in multiple modalities (e.g., visual and auditory), in background noise (with Hua et al. (2014) reporting background noise negatively affects perceived exertion regardless of hearing status), and/or for longer durations (completion time of VOLT in the present study averaged 14 min, ranging from 9 to 22 min). While such additions could increase task complexity, it could also increase the number of variables potentially confounding the ability to identify direct associations (if any) between tinnitus and cognition in office-like tasks.

**FIGURE 3:**
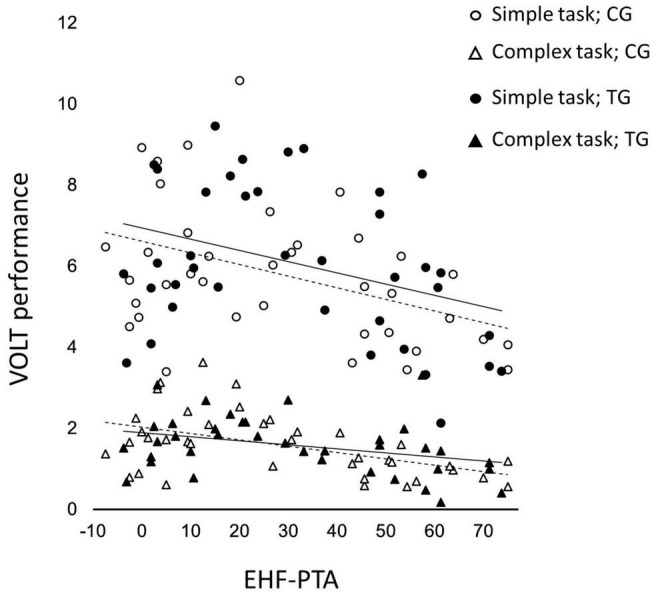
Association between VOLT performance on the simple subtask and complex subtask, and extended hearing frequency thresholds (EHF; 10 to 16 kHz), in tinnitus group (TG) and control group (CG). Upper solid line showing line of best fit for simple subtask in TG, lower solid line showing line of best fit for complex subtask in TG, upper dashed line showing line of best fit for simple subtask in CG, lower dashed line showing line of best fit for complex subtask in CG.

While anxiety, depression, S-PTA and EHF-PTA were all used as covariates in the present study, the finding that only the corrections for EHF-PTA were significant when analyzing tinnitus and VOLT performance (regardless of task complexity) warrants further discussion (see Figure 3 for visual presentation of the association between VOLT and EHF-PTA). This finding suggests that EHF-PTA (and not tinnitus) was associated with performance on an everyday activity that engaged multiple cognitive domains. This association was consistent with Waechter et al. (2019, 2021) who reported that EHF-PTA (and not tinnitus) was associated with performance on laboratory measures of working memory in a similar sample of adults. To further explore the relationship between hearing above 8 kHz and cognitive performance, we conducted partial correlation calculations between EHF-PTA and VOLT performance (simple, complex and total task) controlling for age. Significant negative correlations were found for all task conditions, indicating that higher hearing thresholds above 8 kHz (i.e., poorer high frequency hearing) was associated with lower VOLT scores (i.e., poorer cognitive performance) regardless of age. It should, however, be noted that the significant partial correlations were weak, meaning that the variance in high frequency hearing only accounted for a small amount of variation in cognitive performance.

There are several current hypotheses regarding the driving factor explaining associations between hearing and cognitive performance (Uchida et al., 2019), all of them being relevant and plausible. Several of these hypotheses suggest a causal relationship, where decreased auditory function has negative impact on cognitive function. Examples include the cognitive load hypothesis suggesting greater cognitive resources are required to understand speech in the presence of hearing impairment which in turn limits ones available working memory capacity (Sweller et al., 2011), and the cascade hypothesis suggesting decreased sensory stimulation of the brain reduces total brain volume (Lin et al., 2014; Golub, 2017) and thereby limits resources needed for cognitive operations. There are, however, also hypotheses of a non-causal relationship, such as the common cause hypothesis suggesting a common underlying mechanism between hearing impairment and cognitive decline (Stahl, 2017).

Out of these hypotheses we postulate that the observed associations between high frequency hearing and cognitive performance could be better explained by the common cause hypothesis or the cascade hypothesis. The cognitive load hypothesis (suggesting greater cognitive resources are required to understand speech in the presence of hearing impairment) would initially seem less likely given the importance of hearing in the mid-frequencies (0.8 to 4 kHz) for speech intelligibility and the corrections for S-PTA not being significant in the present study. The potential for hearing loss in the EHFs (above 8 kHz) to contribute to cognitive load should not be rejected outright, however, given recent suggestions that EHF hearing could be needed for optimal speech intelligibility in challenging listening situations (Yeend et al., 2019; Hunter et al., 2020; Trine and Monson, 2020) and the corrections for EHF-PTA being significant in the present study.

The findings of correlations between hearing thresholds above 8 kHz and VOLT performance regardless of task complexity when controlling for age may spark speculations regarding a range of possible implications for clinic, research and future development of hearing aids. Future studies are needed to determine which of the above mentioned hypotheses are at play and to what degree they explain the relationship between auditory and cognitive function. These studies could include longitudinal measures of the relationship between high frequency hearing and cognitive performance, while controlling for aspects such as simultaneous auditory event related potentials and cerebral atrophy (to give an indication whether the cascade hypothesis may be at play), and/or cardiovascular function (Yoshioka et al., 2010; Livingston et al., 2017) and markers of oxidative stress (Cobley et al., 2018) (to give an indication whether the common cause hypothesis may be at play). In clinical settings, measuring hearing thresholds above 8 kHz should already be implemented in the standard audiometric test battery as it adds minimal test time and could help clinicians in the early identification of individuals in need of interventions to mitigate potential decline in cognition (Waechter et al., 2019, 2021) and speech intelligibility (Yeend et al., 2019; Hunter et al., 2020; Trine and Monson, 2020). It seems as if auditory stimulation using hearing aids could be a promising candidate for an intervention in response to high frequency hearing impairment. Recent research has indicated adequately fitted hearing aids to have positive impact on neural reorganization leading to reversal of the cross-modal reorganization between the auditory and visual cortex (Glick and Sharma, 2020), mitigation of tinnitus (Simonetti et al., 2022; Waechter and Jönsson, 2022), as well as improved cognitive function and speech intelligibility (Glick and Sharma, 2020). However, today’s hearing aids rarely deliver amplification above about 10 kHz. The recent findings indicating disadvantages related to decreased auditory function at 10–16 kHz indicates that the benefits of using hearing aids could hypothetically be expanded if the frequency response of future hearing aids could extend to these higher frequencies.

While the correction for EHF-PTA was significant in the analysis of task performance, it was not significant for the analysis of perceived exertion. In line with this finding was the absence of significant correlations between performance on the VOLT and subjective ratings of exertion during the VOLT in all participants or in participants by group (TG or CG). This was consistent with Jahncke and Halin (2012) and Hua et al. (2014) who reported perceived exertion is dissociated from performance on cognitive tasks, with each being influenced by different factors. This suggests that an individual’s performance on an office-like task is not a direct consequence of exertion, with performance and exertion each relating to different aspects of cognition.

### Future Research

Future research investigating potential associations between tinnitus and performance and exertion in office-like tasks should consider at least two factors. The first factor is the inclusion of more objective measures of cognitive exertion (such as pupillometry: van der Wel and van Steenbergen, 2018). Objective measures would be favorable as the connection between subjective and objective measures of exertion have shown to be weakened in adults completing listening and physical tasks simultaneously (Boutcher and Trenske, 1990; Potteiger et al., 2000). The second factor is the need to increase the complexity of the office-like task to induce greater levels of fatigue, particularly by adding background noise typical of office settings (e.g., low level ventilation sounds).

### Limitations

An important aspect to consider when expected significant findings are absent is the statistical power of the analysis. Without information regarding the statistical power it is difficult to interpret whether a meaningful difference is not present or whether it is just not detected. In order to address this issue, we performed a post-hoc power analysis using the G*Power 3.1.9.7 software (Faul et al., 2007, 2009) to compute achieved power. For our MANOVA of VOLT performance (simple, complex, and total task performance), the post-hoc power analysis indicated that our sample size of 76 participants divided into two groups had a statistical power (1-β error probability) of 0.8, when searching for medium sized effects (Cohen’s *f*^2^ = 0.15) and setting the α-level to 0.05. 80% power is the typical power researchers accept when calculating required sample size, why we conclude that the present study was sufficiently powered to detect medium sized effects between the TG and the CG. As for the number of covariates used, we adopted the formula for limit of covariates suggested by Huitema (1980):


C=(0.1*N)-(J-1)

where *C* is the maximum number of covariates for stable estimation of adjusted, *N* is total sample size, and *J* is number of groups. For our study design, Huitema’s (1980) formula indicates that six is the limit of covariates, meaning that our inclusion of four covariates does not imply unstable estimation of adjusted means in our model. While it should be noted that this does not rule out any potential differences in task performance between the groups, if such differences exist the effect size is likely to be small and its clinical relevance would be unclear.

It should also be noted that the included set of participants is not entirely representative of the general tinnitus population. In terms of tinnitus severity, all categories (no handicap, mild handicap, moderate handicap and severe handicap) were represented and with fairly even distribution, though no or moderate handicap were more common than mild or severe handicap among the included participants. This could be compared to the distribution reported by the developers of the THI (Newman et al., 1998), reporting similar, yet more even, distribution of tinnitus handicap. Previous studies have reported differences in cognitive performance between individuals with and without tinnitus regardless if the recruitment strategy has been oriented toward individuals with higher (e.g., Rossiter et al., 2006) or lower degrees (e.g., Jackson et al., 2014) of tinnitus severity. In addition, evidence for the assumed relationship between tinnitus severity and cognitive performance has been mixed. Some studies have reported significant correlations between self-reported tinnitus severity and measured cognitive performances (e.g., Cuny et al., 2004; Jackson et al., 2014), while others have been unable to confirm this (e.g., Dornhoffer et al., 2006; Heeren et al., 2014; Cardon et al., 2019). Taken together, given the typical distribution of tinnitus severity among the included participants of the present study, and the unclear association between tinnitus severity and cognitive performance in previous studies, we conclude that the absence of significant differences in VOLT performance between TG and CG in the present study was likely not due to deviating tinnitus severity.

The included tinnitus participants do, however, differ from the general tinnitus population in terms of educational background. Educational level was high among the included tinnitus participants in the present study, all were either current or former university students. In the general population, however, lower educational background may be a risk factor for tinnitus, as individuals with lower educational level are more likely to work in environments where there is a risk of being exposed to excessive noise (Casey et al., 2017), which may induce tinnitus (Axelsson and Prasher, 2000). This discrepancy between the included tinnitus participants and general tinnitus population is of relevance since lower educational background is also associated with poorer cognitive functioning (Falch and Massih, 2011). Hence, it is not possible to determine whether the present findings are also applicable to tinnitus sufferers with lower educational background.

It is also unclear whether the finding of significant association between hearing above, but not below, 8 kHz and cognitive performance could be due to sampling bias. Most of the included participants had no, or mild to moderate, hearing impairment at standard frequencies (0.125–8 kHz). EHF thresholds were poorer than at standard audiometric frequencies, implying that it may be the case that hearing impairment was associated with cognitive performance were sufficient hearing impairment was present, rather than EHF thresholds being more important for cognitive function than hearing thresholds at standard audiometric frequencies.

Furthermore, we did not control for possible impact of received interventions in the present study. Recent studies have reported that hearing aids may be able to slow down (e.g., Maharani et al., 2018), or even reverse (e.g., Sarant et al., 2020) cognitive decline. This implies that hearing aid use may have significantly impacted the results of the present study, if hearing aid use was more prevalent among participants in either of the groups.

## Conclusion

No associations were found between tinnitus and performance and perceived exertion on the VOLT in adults when corrected for hearing thresholds, anxiety, and depression. The correction for extended high frequency average (10, 12.5, 14 and, 16 kHz) hearing threshold was significant for performance but not perceived exertion on the VOLT. Overall, the results showed extended high frequency hearing, but not tinnitus, was associated with every-day cognitive performance.

## Data Availability Statement

The datasets presented in this article are not readily available because the Regional Ethical Review Board in Lund, Sweden has not granted the researchers to share the dataset with others than those directly involved in the research project. Requests to access the datasets should be directed to SW, sebastian.waechter@med.lu.se.

## Ethics Statement

The studies involving human participants were reviewed and approved by Regional Ethical Review Board in Lund, Sweden. The patients/participants provided their written informed consent to participate in this study.

## Author Contributions

SW and JB designed the experiments. SW collected the data and wrote the manuscript with input from WW, MM, and JB. SW, WW, and JB performed the data analysis. SW, WW, MM, and JB interpreted the results. All authors contributed to the article and approved the submitted version.

## Conflict of Interest

The authors declare that the research was conducted in the absence of any commercial or financial relationships that could be construed as a potential conflict of interest.

## Publisher’s Note

All claims expressed in this article are solely those of the authors and do not necessarily represent those of their affiliated organizations, or those of the publisher, the editors and the reviewers. Any product that may be evaluated in this article, or claim that may be made by its manufacturer, is not guaranteed or endorsed by the publisher.
